# Psychological effects of treatment with new oral anticoagulants in elderly patients with atrial fibrillation: a preliminary report

**DOI:** 10.1007/s40520-014-0243-x

**Published:** 2014-06-01

**Authors:** Stefano Fumagalli, Francesca Cardini, Anna T. Roberts, Serena Boni, Debbie Gabbai, Silvia Calvani, Marta Casalone Rinaldi, Stefania Manetti, Francesca Tarantini, Niccolò Marchionni

**Affiliations:** 1 Intensive Care Unit and Geriatric Arrhythmology Unit, Division of Geriatric Cardiology and Medicine, and Research Unit of Medicine of Aging, Department of Experimental and Clinical Medicine, University of Florence and AOU Careggi, Viale Pieraccini, 6, 50139 Florence, Italy; 2 School of Nursing, University of Florence, Florence, Italy

**Keywords:** Anti-Clot Treatment Scale, Atrial fibrillation, Elderly, New oral anticoagulants, Oral anticoagulation, Perceived Stress Scale

## Abstract

**Background and aims:**

Atrial fibrillation (AF) is the most common arrhythmia in elderly people, yet oral anticoagulation is underused in the aged. We tried to determine whether new oral anticoagulants (NOA) have greater psychological tolerability than warfarin.

**Methods:**

Age-, gender-matched groups of AF patients receiving NOA (*N* = 15) or warfarin (*N* = 15) were assessed with the Anti-Clot Treatment Scale (ACTS) and the Perceived Stress Scale (PSS).

**Results:**

Patients were old (81 ± 9 years). NOA group showed greater psychological satisfaction, with lower therapy-related burden (ACTS burdens: 16.3 ± 4.5 vs. 32.9 ± 10.2, *p* < 0.001) and higher awareness of benefits (ACTS benefits: 13.0 ± 1.3 vs. 10.8 ± 1.9, *p* = 0.001). Even stress was lower (PSS: 13.1 ± 4.0 vs. 17.1 ± 4.2, *p* = 0.013). The multivariate analysis confirmed these findings, showing that higher levels of anxiety and depression could justify more stress in warfarin patients.

**Conclusions:**

The results of this preliminary study show that NOA have an improved psychological impact compared with warfarin in elderly patients.

## Introduction

Atrial fibrillation (AF) is the most common sustained arrhythmia in elderly individuals [1]. Epidemiological data and the greater clinical complexity of old AF patients [2] explain the higher age-associated risk of cardioembolic events [3]. AF determines about 25 % of all ischemic strokes [3]. Oral anticoagulation (OA) is highly effective in preventing cardioembolism [1], and is effective and safe also at advanced ages [4]. Current guidelines recommend OA not only for all AF patients but for all those with a history of AF and a CHA_2_DS_2_-VASc score ≥1, even in the presence of sinus rhythm; since an age ≥75 years is worth two points, it follows that all elderly patients should be anticoagulated [1]. However, warfarin is not ideal for elderly patients: its dose is inversely related to age [5]; the time in therapeutic range (TTR) is influenced by cognitive function [5]; falls are associated with an increased incidence of bleedings [4]. New oral anticoagulants (NOA), with their stable and predictable clinical pharmacology, act rapidly, do not need a laboratory monitoring and do not interfere with other drugs or food; therefore, they could represent a real breakthrough for OA in elderly individuals [5].

Aim of this preliminary study was to compare the psychological effects of treatment with NOA and with warfarin in old AF patients. Greater satisfaction and less stress could increase adherence to treatment, reducing the risk of cardioembolism.

## Methods

### Patients

We enrolled all patients who were prescribed a NOA (for AF or for a history of AF, previously treated with external cardioversion—ECV—or catheter ablation) in our Centre from July to September 2013 (in Italy use of dabigatran and rivaroxaban was approved in June and August 2013, respectively). They were age and gender matched with a population receiving warfarin. Exclusion criteria were: (1) clinical instability or modifications of medical therapy in the last 3 months; (2) any attempted cardioversion in the last month; (3) catheter ablation of AF in the last 3 months. All patients gave their informed consent to participate in the study.

Clinical variables, the ability to manage own medication, health-related quality of life (HRQL), as evaluated with the European Quality of Life Questionnaire (EQ-5D-3L) and Visual Analog Scale (EQ-VAS), and the Hospital Anxiety and Depression Scale (HADS) [6] score were considered for each patient.

### Psychological assessment

We assessed all patients after at least 4 weeks of treatment, using the Anti-Clot Treatment Scale (ACTS), which specifically addresses the degree of psychological satisfaction associated with chronic OA, defining the burdens and benefits of treatment [7]. At the same time, we administered the Perceived Stress Scale (PSS), which assesses to which extent patients feel that their life is dominated by incontrollable and unpredictable factors [8].

### Statistical analysis

We used IBM SPSS for Windows (ver. 20.0). Continuous variables are expressed as mean ± SD, categorical variables as raw numbers and percentages. Different distributions of continuous variables were verified with Student’s *t* test and analysis of variance, or with the related non-parametric tests in the case of non-normal variables. The Chi-square test was used for categorical variables. Simple and multivariate linear regression models were employed to assess the independent role of NOA in determining ACTS and PSS scores. Statistical significance was attributed for *p* values <0.05.

## Results

### Study population

We assessed 17 patients receiving a NOA (dabigatran: *N* = 16, rivaroxaban: *N* = 1); two were excluded (dabigatran: *N* = 1, rivaroxaban: *N* = 1) because treatment had been started <4 weeks before. The warfarin group consisted of 15 patients, of which ten in follow-up after ECV of persistent AF. Two other patients, treated with ECV <4 weeks before assessment, were not enrolled because the procedure could bias the tests. Modal duration of OA was 4 months. The prevalence of patients with dominant sinus rhythm (NOA: *N* = 12, 80 % vs. Warfarin: *N* = 12, 80 %, *p* = 1.000) or with permanent AF (NOA: *N* = 3, 20 % vs. Warfarin: *N* = 1, 6.7 %, *p* = 0.598) did not differ between groups. Enrolled subjects were old (mean age: 81 ± 9 years); hypertension and chronic heart failure were the most common comorbidities. Scores of both CHA_2_DS_2_-VASc and HASBLED were high, as was the average number of drugs taken daily (Table 1). Almost all patients were independent in BADL and IADL (93.3 and 76.7 %, respectively).

**Table1:** Characteristics of patients by treatment group

	Warfarin (*N* = 15)	NOA (*N* = 15)	*p*
Age (years)	79 ± 10	84 ± 7	0.158
Men (*n*, %)	11 (73.3)	9 (60.0)	0.700
Higher education (*n*, %)	8 (53.3)	5 (33.3)	0.462
Retired (*n*, %)	13 (86.7)	14 (93.3)	1.000
Married (*n*, %)	10 (66.7)	5 (33.3)	0.143
Living alone (*n*, %)	1 (6.7)	4 (26.7)	0.180
IADL—Drugs (n, %)	12 (80.0)	8 (53.0)	0.245
Bleeding (*n*, %)	4 (26.7)	2 (13.3)	0.651
CHF (*n*, %)	7 (46.7)	6 (40.0)	1.000
Cognitive impairment (*n*, %)	4 (26.7)	6 (40.0)	0.291
CVD (*n*, %)	3 (20.0)	6 (40.0)	0.427
Diabetes (*n*, %)	3 (20.0)	1 (6.7)	0.598
Hypertension (*n*, %)	11 (73.3)	13 (86.7)	0.651
Myocardial infarction (*n*, %)	6 (40.0)	2 (13.3)	0.215
Renal failure (*n*, %)	3 (20.0)	2 (13.3)	1.000
CHA_2_DS_2_-VASc (score)	4.2 ± 1.6	4.4 ± 1.3	0.711
HASBLED (score)	2.9 ± 1.3	3.0 ± 0.8	0.734
Pacemaker (*n*, %)	1 (6.7)	4 (26.7)	0.330
ASA (*n*, %)	3 (20.0)	0 (0.0)	0.224
Thienopyridines (*n*, %)	2 (13.3)	1 (6.7)	1.000
Drugs (*n*)	7.4 ± 3.3	5.9 ± 3.7	0.236

*Higher education* high school diploma or university degree, *IADLDrugs* patients who do not manage therapy by themselves, *CHF* chronic heart failure, *Cognitive impairment* mild cognitive impairment, *CVD* TIA/Stroke, *ASA* aspirin

More warfarin patients had a score over the 70th percentile of HADS total score (46.7 vs. 13.3 %; *p* = 0.046).

### Psychological satisfaction and type of OA

**Fig. 1:**
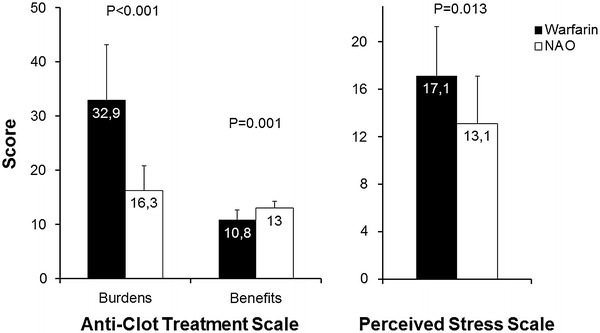
Scores of Anti-Clot Treatment Scale (*left panel*), and of Perceived Stress Scale *(right panel*), by type of oral anticoagulation (*NAO* new oral anticoagulants)

NOA were significantly associated to a higher degree of psychological satisfaction when compared to warfarin. As assessed with ACTS, the psychological burden related to therapy was lower while benefits were higher in subjects receiving NOA (Fig. 1), who showed a higher global score (68.7 ± 4.7 vs. 49.9 ± 10.0, *p* < 0.001). Psychological satisfaction with NOA was greater in all items, except for items 14 (*p* = 0.051) and 15 (*p* = 0.157), which specifically explore feelings of protection and reassurance correlated to OA.

A multivariate regression analysis model (*R* = 0.815, *p* < 0.001) confirmed that ACTS score was significantly higher in the NOA treatment group (+17.8 ± 2.7 vs. warfarin; *p* < 0.001) and lower in patients with a previous hemorrhage (−7.2 ± 3.4 vs. no hemorrhagic event; *p* = 0.043).

### Psychological stress and type of OA

PSS score was lower in NOA than in warfarin patients (Fig. 1).

In univariate analysis, PSS score was higher in those with a previous ECV. The scale was directly related with HADS (*p* < 0.001) and inversely related with HASBLED (*p* = 0.012) and EQ VAS (*p* = 0.032).

A first multivariate linear regression analysis model (*R* = 0.708, *p* < 0.001) confirmed the inverse association between PSS and NOA assumption (−3.4 ± 1.2 vs. warfarin; *p* = 0.010), HASBLED (β = −1.8 ± 0.6; *p* = 0.005) and EQ VAS (β = −0.10 ± 0.04; *p* = 0.019). The introduction into the model (*R* = 0.765, *p* < 0.001) of the variable expressing a HADS score ≥70th percentile (β = +4.4 ± 1.2 vs. <70th percentile; *p* = 0.001) made the use of NOA not significant (*p* = 0.853).

## Discussion

In an elderly AF population, NOA, when compared to warfarin, are more psychologically acceptable. NOA patients’ ACTS scores were significantly higher than warfarin patients’ in 15 of the 17 items. This confirms similarly positive results from the EINSTEIN-DVT study, which randomized 1,472 patients to rivaroxaban or enoxaparin plus vitamin K antagonists for the treatment of deep vein thrombosis not associated with pulmonary embolism [9].

NOA patients also showed lower levels of psychological stress. In a first multivariate model, NOA and higher values of HASBLED and EQ-VAS were associated with lower stress levels. When we introduced the variable related to the presence of an elevated HADS score in the regression, NOA lost their statistical association with PSS. This could be due to several factors. HASBLED is largely influenced by age and several comorbidities common in the elderly, and it has already been proven that patients aged ≥65 years have significantly lower PSS scores [10]. In an AF population HRQL is influenced mainly by the presence of anxiety or depression [11], and in our warfarin-treated group, there was a higher prevalence of patients with a HADS score ≥70th percentile. We can conclude that a possible reason for lower PSS score in NOA patients is NOA treatment’s positive effects on anxiety and depression symptoms [12].

OA is still significantly underused in elderly patients: in the Euro Heart Survey on atrial fibrillation only 56 % AF subjects aged >80 years received Vit. K antagonists [13]. Furthermore, in these patients, OA treatment is often prematurely interrupted after only a few weeks, usually from fear of adverse effects (especially bleeding) [14]. Our results combined with NOAs’ superior safety (a 51 % lower incidence of intracranial hemorrhages than warfarin) and effectiveness (22 % reduction of cardioembolic events vs. warfarin) [15] allow us to hope that the new drugs could expand and maintain prescription of OA in old and very old patients.

### Study limitations

The main limitation of the study is the small sample size. Second, some warfarin patients had received ECV in the past, a factor determining psychological discomfort. However, after adjustment, our results were not altered by the procedure. Third, some NOA patients had already received warfarin in the past, a behavior possibly increasing the beneficial effects of the new drug. Fourth, all NOA patients received dabigatran so we have no data about rivaroxaban or apixaban.

In conclusion, despite these limitations, our findings seem to show that NOA therapy is psychologically better accepted than warfarin in elderly AF patients. Accordingly, compliance to OA should significantly increase, allowing to reduce the incidence of cardioembolic complications, particularly severe in terms of disability and mortality at advanced ages. On this basis, our results can represent a springing board for future studies: by retrospective power analyses (power: 90 %; two-sided type 1 error rate: 0.05) we estimate about 35 patients per group to test our hypothesis in a randomized controlled clinical trial.

## References

[1] Camm AJ, Lip GY, De Caterina R, Savelieva I, Atar D, Hohnloser SH (2012). 2012 focused update of the ESC guidelines for the management of atrial fibrillation: an update of the 2010 ESC guidelines for the management of atrial fibrillation. Developed with the special contribution of the European Heart Rhythm Association. Eur Heart J.

[2] Fumagalli S, Tarantini F, Guarducci L, Pozzi C, Pepe G, Boncinelli L (2010). Atrial fibrillation is a possible marker of frailty in hospitalized patients: results of the GIFA Study. Aging Clin Exp Res.

[3] Go AS, Mozaffarian D, Roger VL, Benjamin EJ, Berry JD, Borden WB (2013). Heart disease and stroke statistics—2013 update: a report from the American Heart Association. Circulation.

[4] Poli D, Antonucci E, Testa S, Tosetto A, Ageno W, Palareti G (2011). Bleeding risk in very old patients on vitamin K antagonist treatment: results of a prospective collaborative study on elderly patients followed by Italian centres for anticoagulation. Circulation.

[5] Deedwania PC (2013). New oral anticoagulants in elderly patients with atrial fibrillation. Am J Med.

[6] Zigmond AS, Snaith RP (1983). The hospital anxiety and depression scale. Acta Psychiatr Scand.

[7] Cano SJ, Lamping DL, Bamber L, Smith S (2012) The Anti-Clot Treatment Scale (ACTS) in clinical trials: cross-cultural validation in venous thromboembolism patients. Health Qual Life Outcomes 10:120. doi: 10.1186/1477-7525-10-120; http://www.hqlo.com/content/pdf/1477-7525-10-120.pdf 10.1186/1477-7525-10-120 PMC3478969 23013426

[8] Cohen S, Kamarck T, Mermelstein R (1983). A global measure of perceived stress. J Health Soc Behav.

[9] Bamber L, Wang MY, Prins MH, Ciniglio C, Bauersachs R, Lensing AW (2013). Patient-reported treatment satisfaction with oral rivaroxaban versus standard therapy in the treatment of acute symptomatic deep-vein thrombosis. Thromb Haemost.

[10] Cohen S, Janicki-Deverts D (2012). Who’s stressed? Distributions of psychological stress in the United States in probability samples from 1983, 2006, and 2009. J Appl Soc Psychol.

[11] Akintade BF, Chapa D, Friedmann E, Thomas SA (2013) The influence of depression and anxiety symptoms on health-related quality of life in patients with atrial fibrillation and atrial flutter. J Cardiovasc Nurs. doi: 10.1097/JCN.0000000000000107. http://journals.lww.com/jcnjournal/pages/articleviewer.aspx?year=9000&issue=00000&article=99748&type=abstract 10.1097/JCN.0000000000000107 24165697

[12] McCabe PJ (2010). Psychological distress in patients diagnosed with atrial fibrillation: the state of the science. J Cardiovasc Nurs.

[13] Fumagalli S, Nieuwlaat R, Tarantini F, de Vos CB, Werter CJ, Le Heuzey JY (2012). Characteristics, management and prognosis of elderly patients in the Euro Heart Survey on atrial fibrillation. Aging Clin Exp Res.

[14] Hylek EM, Evans-Molina C, Shea C, Henault LE, Regan S (2007). Major hemorrhage and tolerability of warfarin in the first year of therapy among elderly patients with atrial fibrillation. Circulation.

[15] Miller CS, Grandi SM, Shimony A, Filion KB, Eisenberg MJ (2012). Meta-analysis of efficacy and safety of new oral anticoagulants (dabigatran, rivaroxaban, apixaban) versus warfarin in patients with atrial fibrillation. Am J Cardiol.

